# Active packaging film based on a green composite: fabrication and performance analysis of pineapple leaf fiber-reinforced PBAT

**DOI:** 10.1039/d5ra10078j

**Published:** 2026-05-08

**Authors:** Yijun Liu, Liangyong Zheng, Fanhui Kong, Yongyue Luo, Huangbing Liang, Shaokai Zhang, Xinghao Tu, Zhanwu Sheng, Gang Chen

**Affiliations:** a Hainan Key Laboratory of Storage & Processing of Fruits and Vegetables, Agricultural Products Processing Research Institute, Chinese Academy of Tropical Agricultural Sciences Zhanjiang 524001 China shengzhanwu100@163.com; b Key Laboratory of Tropical Fruit Biology, Ministry of Agriculture & Rural Affairs, South Subtropical Crop Research Institute, Chinese Academy of Tropical Agricultural Science Zhanjiang 524091 China tuxinghao@126.com papercg@scut.edu.cn; c Quality Department, Beijing United-Food Certification Service Co., Ltd Beijing 100097 China; d State Key Laboratory of Pulp and Paper Engineering, College of Light Industry and Engineering, South China University of Technology Guangzhou 510640 China

## Abstract

To achieve the high-value utilization of pineapple leaf waste and to develop green preservative materials, pineapple leaf fiber ultrafine powder (PLFUP) was prepared through oscillatory ultrafine grinding, and PLFUP/PBAT composite films were subsequently fabricated *via* twin-screw extrusion blending followed by single-screw blow molding. The films were evaluated for surface microstructure, thermal properties, gas barrier performance, and degradability, and their effects on mango preservation were also investigated. The results indicated that the optimal grinding time for PLFUP was 10 minutes, yielding a particle size of 49 µm. As the PLFUP content increased, the surface roughness of the composite films increased, while the glass transition temperature, crystallinity, and melting enthalpy decreased. In contrast, the cold crystallization and melting temperatures rose. Furthermore, both water vapor permeability and oxygen permeability increased significantly, and the biodegradation rate was accelerated. When a composite film containing 3% PLFUP was used as a sealed preservation bag, the mangoes exhibited the fewest surface lesions, along with superior color and gloss, demonstrating the best preservation performance. This study successfully developed a functional packaging film with natural antibacterial activity, tunable permeability, and high biodegradability, offering a novel approach for the resource utilization of agricultural by-products and the advancement of eco-friendly fruit and vegetable preservation technologies.

## Abstract 2

For high-value utilization of pineapple leaf waste, pineapple leaf fiber ultrafine powder (PLFUP) was prepared by oscillatory ultrafine grinding, then blended with PBAT and blow-molded into composite films.

## Graphical abstract

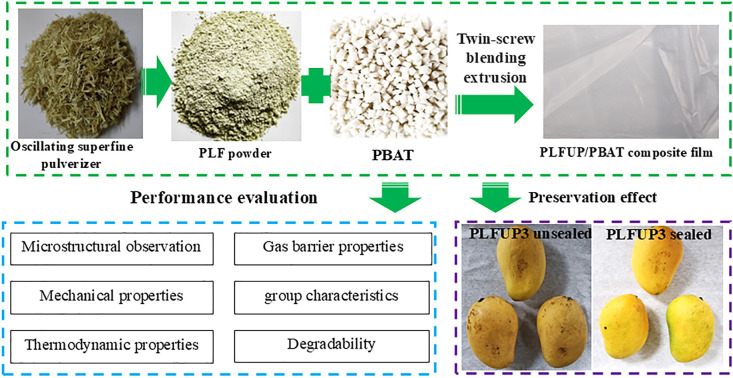

## 1 Introduction

Pineapple is a major tropical fruit in China, with cultivation area exceeding one million acres, playing a vital role in rural revitalization. However, the efficient utilization of its agricultural by-products, particularly pineapple leaves, remains a bottleneck hindering the sustainable development of the industry.^1^ Studies have shown that pineapple leaves contain 1–2% fiber by weight and are rich in bioactive compounds such as phenolics, alkaloids, and triterpenoids, which exhibit antimicrobial properties.^2^ Paper produced from pineapple leaf fiber has demonstrated effective inhibition against common pathogens such as *Staphylococcus aureus* and *Escherichia coli*, highlighting its potential as a functional material.^3^ Currently, conventional pineapple leaf fiber is mainly obtained through semi-automatic mechanical scraping. Although this method yields longer fibers, it involves high labor intensity and production costs (approximately 40 000 RMB per ton), which severely restrict its large-scale application.^4^ In contrast, fiber extracted using pulping and papermaking principles, though shorter in length, offers advantages such as higher production efficiency, lower labor intensity, and significantly reduced cost (≤10 000 RMB per ton). Therefore, it is imperative to overcome the limitations of existing processing technologies, expand high-value applications of pineapple leaf fiber in areas such as packaging, environmental protection, and healthcare, and facilitate its transformation from agricultural waste into functional biomaterials. This will provide scientific support for enhancing the complete value chain and promoting the green and sustainable development of the pineapple industry.

Pineapple leaf fiber, which is rich in bioactive compounds such as phenolics, holds great promise for the development of safe and efficient natural antibacterial preservation materials.^5^ Studies have confirmed that its endogenous phenolic compounds exhibit strong inhibitory effects against a wide range of foodborne pathogens and spoilage microorganisms, including *Escherichia coli*, *Staphylococcus aureus*, *Salmonella*, *Listeria monocytogenes*, *Aspergillus niger*, and *Penicillium*,^6–8^ providing a natural functional basis for constructing broad-spectrum antimicrobial films. Further research has demonstrated that incorporating phenolic compounds such as anthocyanins and gallic acid into composite films can significantly enhance their inhibitory activity against *Botrytis cinerea* in cherries, effectively reduce weight loss, slow down firmness decline, and delay fruit senescence during storage.^9^ Currently, preservation materials are evolving toward multifunctional systems that combine antibacterial activity with modified atmosphere control. For example, a bacterial cellulose/polyvinyl alcohol-based film with excellent oxygen barrier properties (0.3995 cm^3^ m^−2^ 24 h 0.1 MPa), along with water and oil resistance and over 99% antibacterial efficacy, was developed by Li *et al.*,^10^ and apple browning was significantly inhibited. A cellulose-based paper material grafted with guanidine salts and loaded with an ethylene scavenger was constructed by Wei *et al.*,^11^ achieving dual-functional synergy of antibacterial activity and ethylene removal, thereby extending the shelf life of avocados at room temperature to 20 days. An active packaging material based on polycaprolactone/hydroxypropyl methylcellulose nanofibers incorporating carbon dot nanoparticles was developed by Shaghayegh *et al.*,^12^ which demonstrated significant efficacy in meat preservation. Pineapple leaf fiber not only possesses favorable mechanical strength, biodegradability, and processability but is also rich in endogenous antimicrobial compounds such as phenolics, which impart bioactive functionality without the need for external synthetic agents.^2^ As a result, it represents an ideal base material for the development of green and multifunctional active packaging systems.

Poly(butylene adipate-*co*-terephthalate) (PBAT), a biodegradable polyester synthesized from aromatic and aliphatic monomers, exhibits significantly superior flexibility in film form than polylactic acid (PLA) and poly(butylene succinate) (PBS). Its mechanical properties are comparable to those of low-density polyethylene (LDPE), and it undergoes complete degradation in natural environments, rendering it highly promising for eco-friendly packaging applications.^13–16^ However, the inherent lack of antimicrobial activity in PBAT necessitates the incorporation of additives such as zinc oxide and cinnamon essential oil to impart antibacterial functionality for fresh-keeping applications.^17–19^ This not only increases costs but may also raise concerns regarding material safety and degradation behavior. Furthermore, its relatively long complete degradation cycle (approximately 5 months) somewhat restricts its broader application.

Confronted with the dual challenges of low-value agricultural by-product disposal and pollution from non-biodegradable plastics, coupled with the safety risks associated with the heavy reliance on chemical additives in conventional preservation technologies, there is an urgent need to develop integrated solutions. To address these issues, the present study proposes an innovative strategy: converting agricultural waste such as pineapple leaves into high-surface-area, highly reactive PLFUP *via* an ultrafine grinding technology, and incorporating it as a natural antibacterial filler into PBAT. This approach not only enables the high-value utilization of agricultural waste but also successfully yields a biodegradable preservation film with intrinsic antimicrobial activity. The resulting novel material significantly reduces dependence on chemical additives while maintaining favorable biodegradability, thereby providing a new material foundation and technical pathway for the development of an environmentally friendly fruit and vegetable preservation system.

## 2 Materials and methods

### 2.1 Materials

Pineapple leaf fiber (PLF) was purchased from the Institute of Agricultural Machinery, Chinese Academy of Tropical Agricultural Sciences. The “Tainong” mango variety was acquired from Xiashan Comprehensive Vegetable Market in Zhanjiang City. PBAT (TH801T; melt flow rate: 3.1 g/10 min; density: 1.25 g cm^−3^) was obtained from Xinjiang Blue Ridge Tunhe Polyester Co., Ltd.

### 2.2 Preparation of pineapple leaf fiber ultrafine powder

#### 2.2.1 Preparation of powder

PLF was dried in an oven at 60 °C for 12 h and then ground using a vibratory ultrafine grinder (WZJ-12B1, Ji'nan Beili Technology Engineering Co., Ltd, Ji'nan, China) at 4 °C for 5, 10, 15, 20, and 25 min to obtain PLF powders, which were designated as PLF5, PLF10, PLF15, PLF20, and PLF25, respectively.

#### 2.2.2 Measurement of particle size

The particle size of the prepared PLF powders was determined using a laser particle size analyzer (LT2200E, Zhuhai TrueOptics Instrument Co., Ltd, Zhuhai, China). Approximately 2 g of each powder sample was stirred uniformly in a beaker and then transferred into the sample cell of the instrument for measurement, ensuring that the sample concentration remained within the obscuration limits of the instrument. The measurement parameters were as follows: three replicate measurements were performed; the obscuration limits were selected in advanced mode, with a nanoparticle range of 3–7% and a general range of 5–15%. Wet dispersion was used as the measurement method.

### 2.3 Preparation of PLFUP/PBAT composite films

#### 2.3.1 Preparation method of PLFUP/PBAT composite film

PLFUP/PBAT composite films were prepared following the method described by Zhao *et al*.^20,21^ PBAT resin and pineapple leaf fiber ultrafine powders (PLF25) were melt-blended using a twin-screw extruder with a screw diameter of 23 mm and a length-to-diameter ratio of 40 : 1. The PLF25 loadings were set at 0%, 0.5%, and 3% by weight. The barrel temperature profile ranged from 140 °C to 170 °C, and the screw speed was maintained at 150 rpm. The extrudate was subsequently cooled in a water bath, pelletized, and dried to obtain pellets designated as PLFUP0, PLFUP0.5, and PLFUP3.

A portion of the pellets was compression-molded into sheets for mechanical and other property evaluations. Hot pressing was conducted at 160 °C under a pressure of 20 MPa. Sheets with a thickness of 0.5 mm were used for gas barrier testing, while 1 mm-thick sheets were prepared for tensile testing. The remaining pellets were processed into films by blow molding using a single-screw extruder equipped with a screw diameter of 25 mm and a length-to-diameter ratio of 33. The die had an outer diameter of 25 mm and a gap of 1.5 mm. The temperature profile from the feed zone to the die was set at 140 °C/160 °C/160 °C, with the die temperature maintained at 160 °C. The blow-up ratio was controlled between 3.0 and 4.0, and the take-up ratio ranged from 6.0 to 8.0.

#### 2.3.2 Microstructural observation of PLFUP/PBAT composite films

The microstructure of the PLFUP/PBAT composite films was observed according to the method of Zhao *et al*.^20,21^ The surface morphology of the films was analyzed using scanning electron microscopy (SEM). Three types of PLFUP/PBAT composite film samples were mounted on sample stubs using conductive tape and subsequently sputter-coated with a thin layer of gold in a vacuum coater. The samples were then examined and imaged under SEM at accelerating voltages of 5.0 kV and 15.0 kV, with a magnification of 300×.

#### 2.3.3 Mechanical properties test of PLFUP/PBAT composite films

The mechanical properties of the PLFUP/PBAT composite films were tested according to the method of Zhao *et al*.^20,21^ The physical and mechanical properties of the films were measured using a computer-controlled electronic universal testing machine (E43.104, MTS Industrial Systems (China) Co., Ltd). The testing parameters were as follows: a tensile speed of 500 mm min^−1^, a gauge length of 50 mm, a sample width of 10 mm, and film thickness based on actual measurements (ranging from 15 to 30 µm). Eight parallel specimens were prepared for each group: strip-shaped samples (15 cm × 1 cm) for tensile testing and rectangular samples with a notch for tear testing, both with a gauge length of 50 mm and a width of 10 mm. The reported data represent the average values of the measurements.

#### 2.3.4 Differential scanning calorimetry (DSC) of PLFUP/PBAT composite films

The thermodynamic properties of the PLFUP/PBAT composite films were measured according to the method of Zhao *et al*.^20,21^ A differential scanning calorimeter (DSC 214, NETZSCH, Germany) was used to determine the glass transition temperature (*T*_g_) and melting point (*T*_m_) of the blended materials. The measurements were performed under a nitrogen atmosphere with the following temperature program: the sample was heated from room temperature to 180 °C at a rate of 10 °C min^−1^ and held isothermally for 2 min to erase thermal history. It was then cooled to −60 °C at 10 °C min^−1^ and held for 2 min, followed by reheating to 180 °C at 10 °C min^−1^. The data from the second heating cycle were recorded and used for analysis.

#### 2.3.5 Gas barrier property testing of PLFUP/PBAT composite films

The water vapor, oxygen, and carbon dioxide transmission rates of the PLFUP/PBAT composite films were measured according to the methods described by Zhao *et al*.^20,21^ The water vapor transmission rate (WVTR, g (m^−2^ 24 h)) was determined using a water vapor transmission rate tester in accordance with ISO 15106-2 (infrared sensor method) at 23 °C. During testing, water vapor diffused from the wet chamber (maintained at 60% relative humidity) through the sample into the dry chamber. The vapor was then carried by a dry nitrogen purge gas stream to an infrared sensor for detection. The test area was 4 cm^2^, with the dry side maintained at 30% relative humidity and the temperature controlled at 23 °C. The water vapor permeance (WVP, g µm (m^−2^ 24 h)) was calculated as WVP = WVTR × *e*, where *e* (mm) represents the film thickness.

The oxygen and carbon dioxide transmission rates were measured in accordance with the Chinese national standard GB/T 1038.1-2022, Plastics—Determination of gas transmission through films and sheets—Part 1: differential-pressure method.

#### 2.3.6 Degradation test of PLFUP/PBAT composite films

The biodegradation performance of the PLFUP/PBAT composite films was evaluated using a soil burial method adapted from Zeng *et al.*^22^ and Marques *et al.*,^23^ with minor modifications. Film samples were cut into 80 mm × 80 mm squares, dried to a constant weight, and buried approximately 10 cm below the soil surface in natural soil collected from a banana orchard. To maintain optimal degradation conditions, the soil was watered twice daily to ensure consistent moisture content. Every 5–10 days, samples were retrieved, gently rinsed with distilled water to remove soil residues, and dried at 60 °C until a constant weight was achieved. For each group, three parallel samples were tested, and the results were averaged. The degradation rate (*Y*, %) was calculated as *Y* = (*W*_i_ − *W*_*n*_)/*W*_i_ × 100%, where W_i_ and *W*_*n*_ represent the initial mass (g) and the mass at day *n* (g), respectively.

### 2.4 Evaluation of the preservation effect of PLFUP/PBAT packaging bags

Mangoes that were free from mold, uniform in size, and without visible mechanical damage were selected, washed with distilled water, and then air-dried to remove surface moisture. The mangoes were randomly divided into seven groups: three groups were sealed in PLFUP0, PLFUP0.5, and PLFUP3 films; another three groups were stored with the same films but left unsealed; and one group was left unpackaged as a blank control. All samples were stored at room temperature (15 °C) for 7 days. Visual quality changes in the mangoes were observed every day, and weight loss was measured periodically. The effect of different treatments on mango weight loss was evaluated according to Akhter *et al*.^24^ The weight loss rate (*Y*, %) was calculated using *Y* = (*m*_0_ − *m*_*n*_)/*m*_0_ × 100%, where *m*_0_ and *m*_*n*_ represent the initial mass (g) and the mass on day *n* of storage (g), respectively.

### 2.5 Statistical data analysis

All experiments were independently repeated three times, and the results were expressed as mean ± standard deviation. Raw data were organized and preliminarily processed using Microsoft Excel 2019. One-way analysis of variance (ANOVA) was performed using SPSS 22.0, followed by Duncan's multiple range test to determine significant differences at *p* < 0.05, ensuring the statistical validity and reliability of the results. Graphs and figures were generated using OriginLab Origin 2022, with clear visuals and standardized annotations to facilitate data visualization and interpretation. This standardized data processing workflow effectively ensured the accuracy of the experimental data and the credibility of the conclusions, providing a solid statistical foundation for result analysis.

## 3 Results and discussion

### 3.1 Effect of different grinding times on the particle size of pineapple leaf fiber powder

Conventional blade-type or hammer mills often struggle to effectively process fibrous materials with high flexibility. In contrast, an oscillating ultrafine grinder utilizes vibration-driven motion, subjecting the material within the grinding chamber to high-speed impacts, grinding, and friction from stainless steel grinding rods, enabling efficient size reduction of fibers to the micron level.^25,26^ In this study, this equipment was employed for the micronization of pineapple leaf fibers, as shown in Fig. S1. The effect of different grinding durations on the particle size of pineapple leaf fiber powder is presented in Table 1. As indicated by the table, when the grinding time increased from 5 min to 25 min, the particle size significantly decreased from 124.5 µm to 31 µm. Specifically, after 10 min of grinding, the particle size of the pineapple leaf fibers reached 49 µm, achieving micronization. Statistical analysis revealed that differences in D10 and D50 particle sizes across various grinding times were all statistically significant. For the D90 range, grinding durations up to 15 min had a highly significant effect on particle size. However, further extending the grinding time resulted in no significant differences between the particle sizes obtained at 15 min and 20 min, or between those at 20 min and 25 min. The micronized fiber particles exhibit physicochemical properties similar to their bulk counterparts; however, smaller particle sizes lead to larger specific surface areas and enhanced surface activity, which can easily cause particle agglomeration due to intermolecular attraction.^27,28^ Furthermore, studies have shown that once fibers are reduced to the micrometer scale (1–50 µm), they can impart high strength and stiffness when used as fillers in plastics, indicating broad application potential in fields such as agriculture and environmental protection.^29,30^ Therefore, the pineapple leaf fiber microparticles prepared in this study hold considerable promise for future applications.

**Table 1:** Particle size characteristics of pineapple leaf fiber powders^a^

Sample	D10/µm	D50/µm	D90/µm	Residual	Shading degree
PLF5	5.90 ± 0.09 a	27.07 ± 0.39 a	124.5 ± 5.26 a	0.885	11.388
PLF10	4.78 ± 0.01 b	16.36 ± 0.08 b	49.43 ± 0.26 b	0.659	12.042
PLF15	4.23 ± 0.02 c	14.27 ± 0.05 c	37.49 ± 0.19 c	0.624	12.103
PLF20	3.88 ± 0.01 d	13.41 ± 0.02 d	33.83 ± 0.01 cd	0.478	12.423
PLF25	3.57 ± 0.00 e	12.71 ± 0.03 e	31.98 ± 0.07 d	0.562	11.628

a Note: different letters within the same column indicate significant differences at the 0.05 level.

### 3.2 Effect of PLFUP content on microstructure of PLFUP/PBAT composite films

**Fig. 1:**
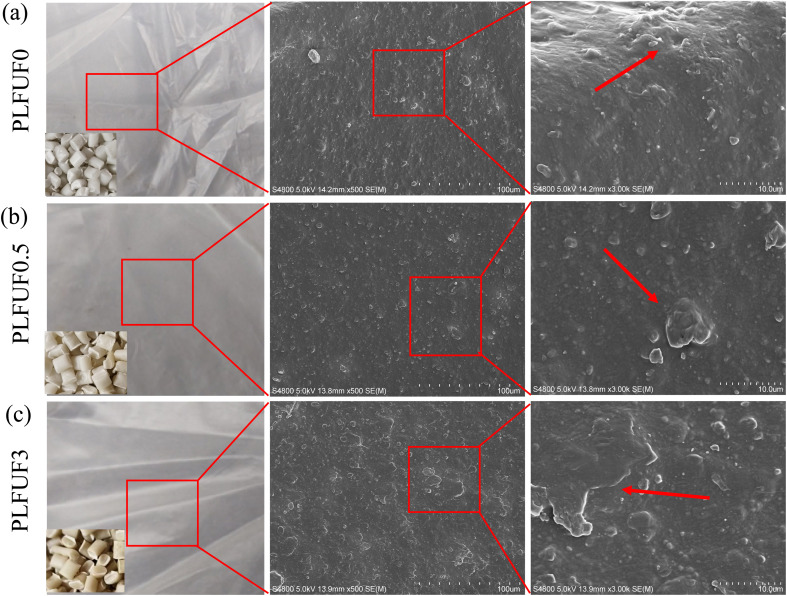
SEM images of PLFUP0 (a), PLFUP0.5 (b) and PLFUP3 (c).

PLFUP/PBAT composite films were fabricated through a process of twin-screw extrusion blending and subsequent single-screw blow molding, as depicted in Fig. 1. The color of the films was observed to change from white to dark green with increasing PLFUP content, a progression attributed to the natural pigments in the fibers and their cumulative presence in the matrix. Distinct granular protrusions were observed on the surfaces of films containing PLFUP, which became more pronounced at higher filler loadings and were primarily ascribed to particle agglomeration. Despite this, the film surfaces maintained relatively good gloss. Conventional plant fibers, typically characterized by large size and high surface polarity, are known for poor dispersion in hydrophobic polymer matrices, limiting their use in films.^31^ Micronized fiber powder, however, is reported to retain the reinforcing effect of natural fibers while significantly enhancing dispersion and interfacial compatibility, thereby supporting the processing and performance of film materials^32^ and indicating considerable potential for industrial applications.

### 3.3 Effect of PLFUP content on mechanical properties of PLFUP/PBAT composite films

The influence of varying loading levels of PLFUP on the mechanical properties of PLFUP/PBAT composite films is shown in Table 2. As shown in Table 2, when the PLFUP content increased from 0% to 0.5%, the tensile strength in the machine direction (MD) was enhanced from 27 MPa to 28 MPa, while the elongation at break was raised from 414% to 560%. However, a further increase in PLFUP content from 0.5% to 3% resulted in a slight decline in both MD tensile strength (from 28 MPa to 27 MPa) and elongation at break (from 560% to 550%), indicating that higher filler loadings did not contribute to the improvement of these properties. Over the entire range from 0% to 3% PLFUP, the transverse direction (TD) tensile strength was reduced from 31.6 MPa to 23.9 MPa, and the TD elongation at break decreased from 701% to 671%. Regarding tear strength, a trend of initial decrease followed by an increase was observed in both MD and TD directions with rising PLFUP content. Specifically, as the PLFUP loading was raised from 0.5% to 3%, the tear strength increased from 132.5 kN m^−1^ to 135.0 kN m^−1^.

**Table 2:** Mechanical property analysis of PLFUP/PBAT composite films

Sample	Tensile strength/MPa	Elongation at break/%	Tear strength/KN m^−1^
PLFUP0-MD	27.0 ± 3.8	414 ± 76	142.6 ± 5.9
PLFUP0-TD	31.6 ± 3.6	701 ± 50	148.1 ± 10.1
PLFUP0.5-MD	28.1 ± 2.6	560 ± 64	94.8 ± 9.6
PLFUP0.5-TD	29.1 ± 2.8	681 ± 28	132.6 ± 9.2
PLFUP3-MD	27.1 ± 3.1	550 ± 43	95.6 ± 3.3
PLFUP3-TD	23.9 ± 2.3	671 ± 36	135.0 ± 9.0

### 3.4 Effect of PLFUP content on the thermodynamic properties of PLFUP/PBAT composite films

**Fig. 2:**
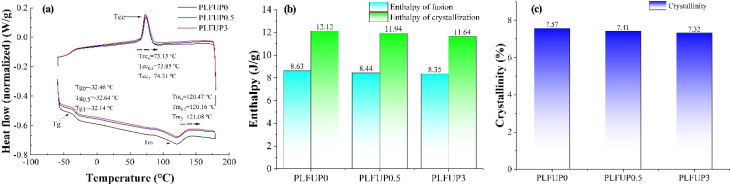
DSC curves of PLFUP/PBAT composite films (a). Enthalpy (b) and crystallinity (c) of the PLFUP degradable film.

The influence of different PLFUP loading levels on the thermal properties of PLFUP/PBAT composite films is shown in Fig. 2. As illustrated, as the PLFUP content increased from 0% to 3%, the crystallinity of the composite films decreased from 7.57% to 7.32%, the crystallization enthalpy decreased from 12.12 J g^−1^ to 11.64 J g^−1^, and the melting enthalpy decreased from 8.63 J g^−1^ to 8.35 J g^−1^. Concurrently, the cold crystallization temperature (*T*_cc_) was observed to increase from 73.13 °C to 74.31 °C, and the melting temperature (*T*_m_) rose from 120.47 °C to 121.08 °C. These changes suggest that the addition of PLFUP restricted the free mobility of the PBAT molecular chains, thereby requiring them to overcome a higher energy barrier to achieve motion.^33^

### 3.5 Effect of PLFUP content on the gas barrier properties of PLFUP/PBAT composite films

**Fig. 3:**
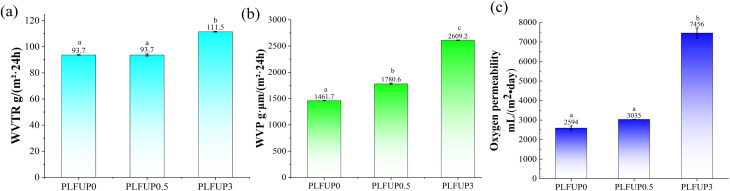
Water vapor transmission rate (a), water vapor permeability (b) and oxygen permeability (c) of PLFUP/PBAT composite films.

The primary function of freshness packaging materials is to regulate the gas concentration inside the package and isolate the external environment. This inhibits fruit respiration and microbial growth, delays metabolic processes, and ultimately prolongs the freshness period.^11,34,35^ Consequently, the water vapor transmission rate (WVTR) and oxygen transmission rate (OTR) of packaging materials are critical for fruit preservation: the former influences the control of moisture loss, while the latter directly affects the regulation of respiratory activity. In this study, all films were prepared with a uniform thickness of 20 µm. The water vapor transmission rate, water vapor permeability coefficient, and oxygen transmission rate of the films are shown in Fig. 3(a–c), respectively. As the PLFUP content increased from 0% to 3%, the WVTR rose from 93.7 to 111.5 g (m^−2^ 24 h) (an increase of 19%), the water vapor permeability coefficient increased significantly from 1461.7 to 2609.2 g µm (m^−2^ 24 h) (a rise of 78%), and the OTR showed a marked increase from 2594 to 7456 mL (m^−2^ day) (a growth of 187%). Statistical analysis revealed that films containing 3% PLFUP loading exhibited statistically significant differences in gas barrier properties compared with both the control group and the 0.5% PLFUP group. This phenomenon could be attributed to the introduction of plant fibers, which altered the microstructure of the film, thereby enhancing its gas permeability. The improved breathability facilitates the dissipation of water vapor and mitigates the accumulation of condensate and gases such as carbon dioxide. This modification disrupts the growth environment of anaerobic microorganisms, suppressing their proliferation and ultimately contributing to an enhanced preservation effect.^36^

### 3.6 Effect of PLFUP content on the degradability of PLFUP/PBAT composite films

**Fig. 4:**
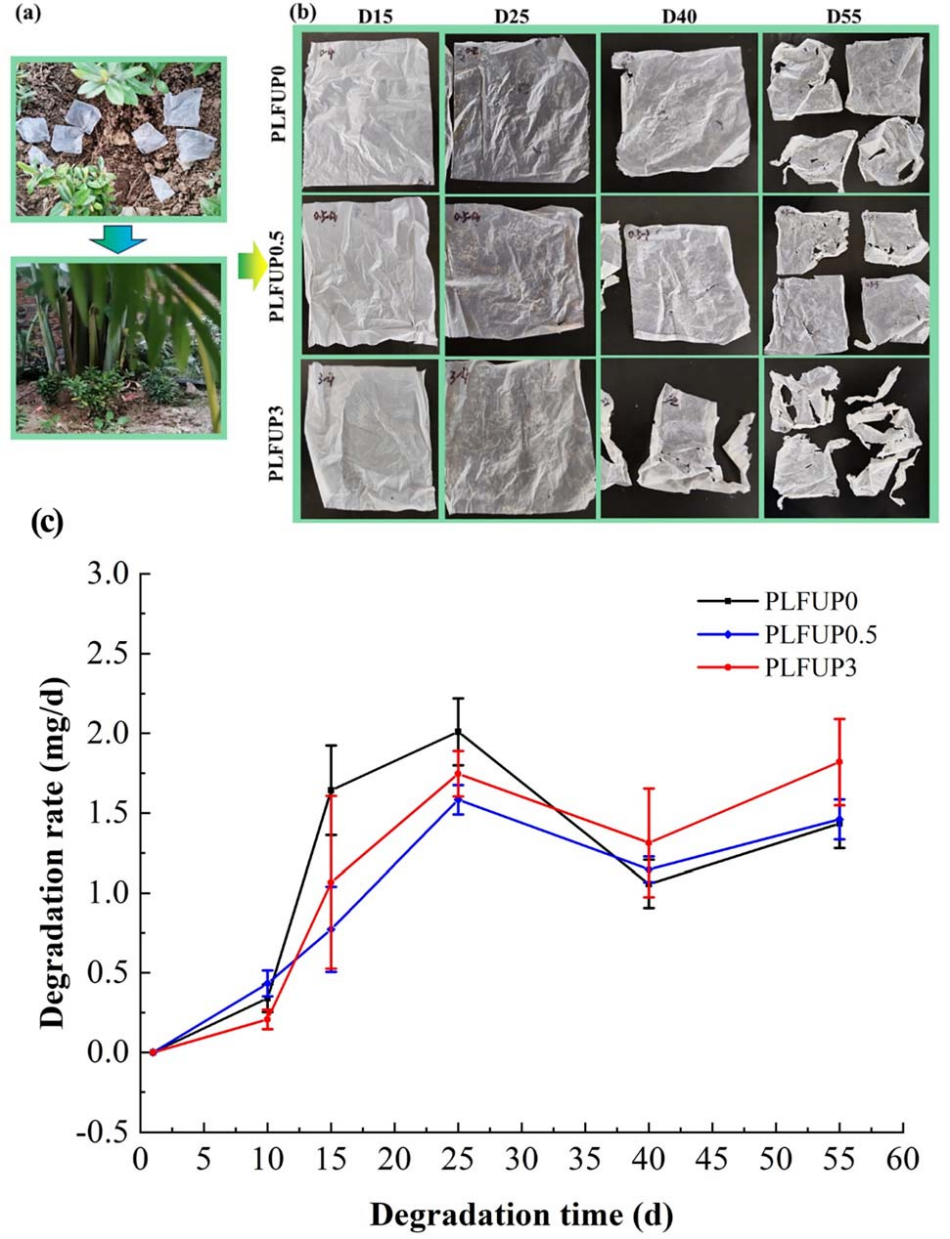
Degradation of PLFUP/PBAT composite films in an outdoor soil environment (a), degradation behavior (b) and degradation rate (c).

The degradation environment, degradation performance, and degradation rate of PLFUP/PBAT composite films in soil are shown in Fig. 4(a–c), respectively. As illustrated in Fig. 4(b), distinct differences were observed in the degradation behavior of films with varying contents of PLFUP. In the initial stage of degradation (25 days), the shapes of all film groups remained largely intact. By day 40, films in the PLFUP3 group began to become brittle, with the appearance of a small number of fragments and holes. By day 55, the extent of degradation had increased significantly, displaying clear degradation characteristics. In contrast, the PLFUP0 and PLFUP0.5 groups exhibited only brittleness and a limited number of fragments at day 55.

As shown in Fig. 4(c), the degradation rate of the films displayed a fluctuating trend of “rise–fall–rise” over the 55 day period. Between days 10 and 30, the degradation rates of samples containing PLFUP were lower than those of the control group, which may be attributed to antibacterial effects associated with certain fiber components. After day 40, however, the degradation rates of the fiber-containing films increased markedly, exceeding those of the control group. Furthermore, a strong positive correlation was observed between fiber content and degradation rate. This pattern may be explained by the gradual dissolution or decomposition of antibacterial small-molecule substances within the fibers over time, which likely diminished their inhibitory effect on microorganisms and facilitated microbial colonization and metabolic activity. Additionally, PLFUP is rich in polysaccharides such as cellulose and hemicellulose, which are readily utilized by microorganisms, leading to a biodegradation rate significantly higher than that of pure PBAT.^37^ The incorporation of hydrophilic PLFUP into the hydrophobic PBAT matrix also increases the specific surface area of the material, enhances water penetration and enzyme adsorption, and thereby accelerates both hydrolytic and microbial degradation processes.^38^

### 3.7 Effect of PLFUP content on the preservation of PLFUP/PBAT composite films

**Fig. 5:**
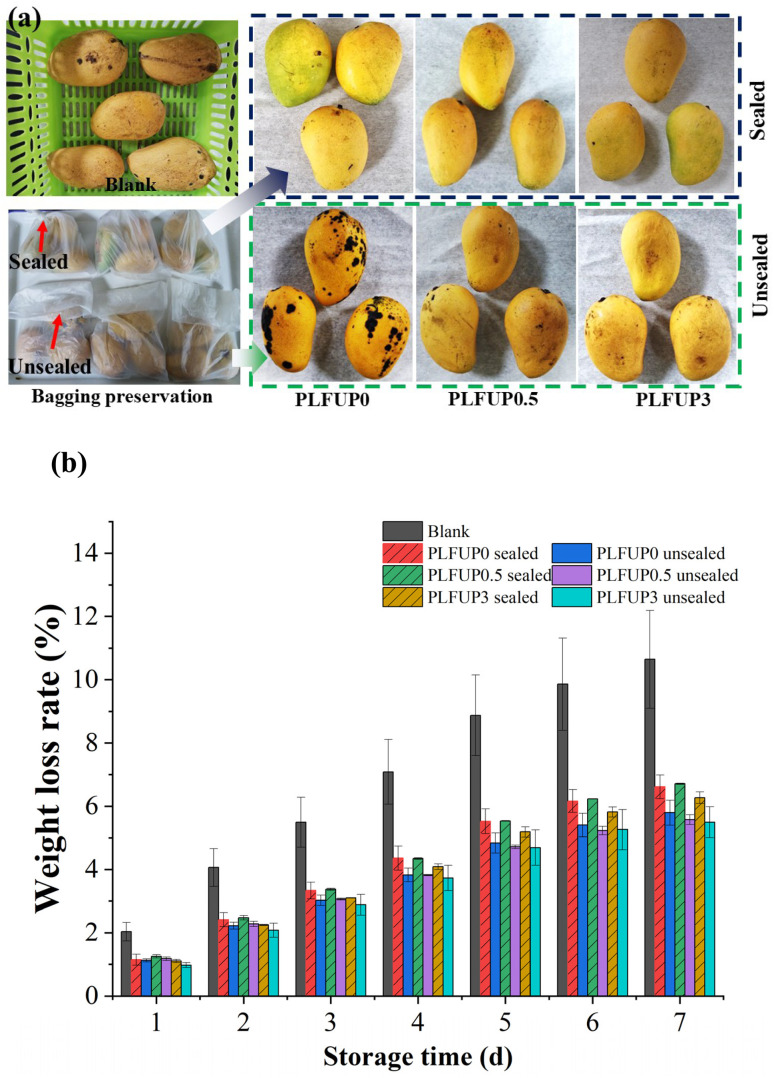
Preservation effects of the PLFUP/PBAT composite film on mangoes (day 7) (a), and the weight loss rate of mangoes at different storage times (b).

To further verify the practical application potential of the prepared films for mango preservation, the films were fabricated into packaging bags, and their effects on the mango storage quality were evaluated, as shown in Fig. 5. As shown in Fig. 5(a), compared with the control group, mangoes packaged in the film bags exhibited improved preservation quality, and sealing the bag opening resulted in even better preservation performance. With increasing PLFUP particle content, the formation of surface lesions on mango peels was noticeably inhibited. When the PLFUP content reached 3%, the mango peel remained smooth and dark green, demonstrating the optimal preservation effect. Previous studies have indicated that the primary pathogen responsible for postharvest mango decay is *Colletotrichum* spp., a strictly aerobic fungus whose growth and pathogenicity are highly dependent on oxygen.^39,40^ This suggests that one of the key mechanisms by which the packaging bags exert their beneficial effects is by modifying the internal microenvironment to suppress the growth of *Colletotrichum* spp.

As shown in Fig. 5(b), the weight loss rate of mangoes stored in bags in all treatment groups increased throughout the storage period. However, mangoes stored in bags treated with the PLFUP/PBAT composite film showed a significantly lower weight loss rate than the control group without the film, indicating that the film effectively reduced water evaporation from the fruit. Although the weight loss rate in the sealed group was slightly higher than in the unsealed group—possibly due to a slight temperature rise inside the sealed bag, which enhanced respiratory metabolism—the sealed packaging significantly limited the contact of mangoes with the external environment, thereby reducing the risk of pathogen infection. As a result, mangoes in the sealed group exhibited better peel gloss and appearance quality, contributing to an overall improved preservation effect. In conclusion, the combination of 3% PLFUP/PBAT composite film with sealing can be considered an effective strategy for mango preservation.

## 4 Conclusion

In this study, PLFUP was successfully prepared through ultrafine grinding technology, and PLFUP/PBAT composite films were subsequently fabricated *via* twin-screw compounding and extrusion. The results indicated that a grinding duration of 10 minutes significantly reduced the fiber particle size. While extended grinding times led to further size reduction, the particle size was observed to stabilize after 20 minutes. With increasing PLFUP content, the surface roughness of the films was enhanced, and notable changes in thermal properties were observed: the glass transition temperature, crystallinity, and melting enthalpy decreased, whereas the cold crystallization and melting temperatures increased. Concurrently, increases in both water vapor and oxygen transmission rates were observed, along with an accelerated biodegradation rate.

In application tests, the film was found to effectively inhibit the infection of the anthracnose fungus and significantly improve the storage quality of mangoes by modifying the micro-environment within the packaging. Specifically, the film with 3% PLFUP addition demonstrated optimal preservation performance under sealed conditions, effectively maintaining the color and surface gloss of the fruit peel. Although sealed packaging was noted to slightly exacerbate moisture loss, its superior efficacy in suppressing disease development was more prominent. In conclusion, the PLFUP/PBAT composite films exhibited satisfactory biodegradability and effective preservation functionality, offering a viable approach for the high-value utilization of agricultural by-products and the development of eco-friendly functional food packaging materials.

## Conflicts of interest

There are no conflicts to declare.

## Supplementary Material

RA-016-D5RA10078J-s001

## Data Availability

The data that support the findings of this study are available from the corresponding author upon reasonable request. Supplementary information (SI): schematic diagram and observed effects of ultrafine grinding of pineapple leaf fiber. See DOI: https://doi.org/10.1039/d5ra10078j.
