# Supplementary material for: Active packaging film based on a green composite: fabrication and performance analysis of pineapple leaf fiber-reinforced PBAT
Source: RSC Adv. 2026 May 8;16(27):24296–305. doi: 10.1039/d5ra10078j (PMC13154777; doi:10.1039/d5ra10078j)
Supplement: RA-016-D5RA10078J-s001 [file RA-016-D5RA10078J-s001.pdf]

Supplementary Information (SI) for RSC Advances.
This journal is © The Royal Society of Chemistry 2026

Supporting Information

Active packaging film from a green composite: Pineapple leaf fiber reinforced PBAT – fabrication and performance analysis

Yijun Liu [1,2], Liangyong Zheng [1], Fanhui Kong [3], Yongyue Luo [1], Huangbing Liang [1], Shaokai Zhang[1], Xinghao Tu [2]*, Zhanwu Sheng [1], Gang Chen[2]*

1 Hainan Key Laboratory of Storage & Processing of Fruits and Vegetables, Agricultural Products Processing Research Institute, Chinese Academy of Tropical Agricultural Sciences, Zhanjiang 524001, China;

2 Key Laboratory of Tropical Fruit Biology, Ministry of Agriculture & Rural Affairs, South Subtropical Crop Research Institute, Chinese Academy of Tropical Agricultural Science, Zhanjiang, 524091, China;

3 Quality Department, Beijing United-Food Certification Service Co., Ltd., Beijing, 100097, China;

4 State Key Laboratory of Pulp and Paper Engineering, College of Light Industry and Engineering, South China University of Technology, Guangzhou 510640, China;

* Correspondence: tuxinghao@catas.cn (Xinghao Tu), shengzhanwu100@163.com (shengzhanwu100@163.com) and papercg@scut.edu.cn (Gang Chen)

Yijun Liu and Liangyong Zheng have contributed equally.

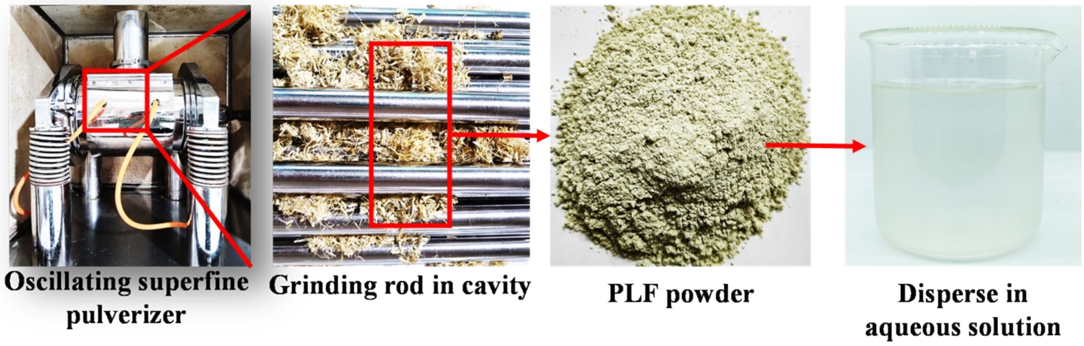

Figure S1. Schematic diagram and effect of ultrafine grinding of pineapple leaf fiber
